# Impact of work instruction difficulty on cognitive load and operational efficiency

**DOI:** 10.1038/s41598-025-95942-7

**Published:** 2025-04-01

**Authors:** Abdulrahman K. Eesee, Vera Varga, György Eigner, Tamás Ruppert

**Affiliations:** 1 Department of System Engineering, University of Pannonia, Veszprém, 8200 Hungary; 2 Institute of Psychology and Mental Health, University of Pannonia, Veszprém, 8200 Hungary; 3 Biomatics and Applied Artificial Intelligence Institute, John von Neumann Faculty of Informatics, Obuda University, Budapest, 1034 Hungary; 4 Physiological Controls Research Center, University Research and Innovation Center, Obuda University, Budapest, 1034 Hungary; 5 HUN-REN-PE Complex Systems Monitoring Research Group, University of Pannonia, Veszprém, 8200 Hungary; 6 Department of Medical Instrumentation Technology Engineering, Northern Technical University, Mosul, 41001 Iraq

**Keywords:** Work instruction, Assembly, Cognitive load, GSR, HRV, CLT, Human behaviour, Biomedical engineering, Computer science

## Abstract

As industries progress toward integrating more complex technologies within Industry 4.0 frameworks, ensuring work instructions that balance cognitive load and performance is increasingly critical, especially under the human-centric principles of the 5th industrial revolution. Drawing on Cognitive Load Theory (CLT), this study compares two instructional methods-visual-based and code-based-to determine whether cognitive overload can be reduced without compromising task outcomes in a controlled, assembly-like scenario derived from industrial tasks. We recruited 30 participants from the academic field (students and researchers), who completed assembly tasks under both visual-based and code-based instructions. Cognitive load was measured objectively by (Galvanic Skin Response, Heart Rate Variability, and hand motion acceleration) and subjectively through (NASA Task Load Index, short Dundee Stress State Questionnaire). Operational efficiency was assessed via task completion time (TCT), number of task repetitions (NTR), and assembly precision based on the standard deviation. The findings demonstrated that visual-based instructions significantly reduced cognitive load with a $p-value <0.001$. It also showed an improvement in two of the performance metrics during the use of visual-based instructions for the TCT and NTR with $p-values <0.001$. However, although code-based instructions increased cognitive load, they showed better assembly precision with a $p-value < 0.001$. These results suggest that while simple and direct instructions facilitate task execution and reduce cognitive loads, deep thinking approaches may still hold value for tasks requiring high precision.

## Introduction

In modern industrial settings, the dynamic nature of the workforce and the rising costs of human labor necessitate implementing efficient and effective training and assembly procedures^1^. The introduction of Operator 4.0, a framework that integrates technological advancements with a human-centric approach, aims to enhance operational efficiency and worker well-being^2^. As industries evolve to embrace more advanced technologies and complex processes, there is a pressing need to ensure that human operators are not only efficient but also resilient and well-supported in their roles^3^. Human operators in these environments face multifaceted challenges, intensified by the rise in product variants that require precise cognitive engagement. Supporting these operators effectively involves not only enhancing the clarity and accessibility of work instructions but also customizing these instructions to reduce cognitive load-a concept grounded in Cognitive Load Theory (CLT)^4,5^. Given these escalating complexities and the imperative for human-centric approaches, re-assessing conventional work instructions emerges as a vital step to maintain productivity, reduce errors, and manage operator strain in increasingly dynamic manufacturing scenarios^4,6^.

In the industrial setting, poorly designed instructions can significantly undermine productivity, increase the likelihood of errors, and lower overall job satisfaction. Moreover, the detrimental economic and social consequences of poor instruction have been extensively documented, resulting in reduced levels of customer satisfaction, increased operational costs, and inefficient decision-making processes^7^. This highlights the necessity for companies to prioritize high-quality information in their operational instructions^7–10^. Although numerous studies have explored the benefits of simplified or digital work instructions-such as textual guides or augmented reality (AR)-based solutions^11,12^-these approaches often do not systematically validate the objective metrics with the subjective experience of workers based on the utilized instructions. Furthermore, research that integrates subjective questionnaires and objective physiological metrics to comprehensively evaluate worker cognitive load and efficiency based on work instructions remains limited. This gap is particularly pressing in modern assembly environments, where rising task complexity calls for instruction designs that are both cognitively considerate and operationally effective.

To address this gap, the present study systematically compares two distinct instructional approaches-code-based and visual-based-within an assembly-like scenario. Specifically, we hypothesize that code-based instructions, which rely on alphanumeric codes to guide the assembly process, impose a higher subjective cognitive load due to the increased mental effort required to decipher the codes. By contrast, visual-based instructions are expected to reduce cognitive load by offering more intuitive, graphical representations of the same tasks. However, this simplified approach may induce more frequent hand movements and repeated task cycles-potentially resulting in more pronounced changes in physiological signals (Galvanic Skin Response GSR and Photoplethysmogram PPG) due to increased physical activity. In evaluating these hypotheses, we measure both subjective cognitive load (using the NASA Task Load Index ‘NASA_TLX’ and short Dundee Stress State Questionnaire ‘short DSSQ’) and objective indicators (physiological signals and task performance metrics) to capture a comprehensive view of how work instructions influence operator well-being and efficiency. We therefore pose the central question: *How do subjective perceptions of cognitive load and performance align with objectively measured changes in cognitive load and performance when different instructional methods are employed?*

The next subsections detail the theoretical and practical frameworks-Cognitive Load Theory and Worker Performance-to further contextualize our research.

### Cognitive load theory (CLT)

CLT serves as the primary framework for assessing the effectiveness of work instructions in this study. Cognitive load refers to the amount of mental resources and effort required to process information and carry out a particular task. It represents the demand placed on working memory during task execution. CLT highlights that while our long-term memory has an expansive capacity, our working memory is significantly more limited. The theory defines three types of cognitive loads, each impacting the efficiency of our information processing. The first type, *”Intrinsic Cognitive Load”*, deals with the degree of complexity associated with the acquisition of new knowledge^5,13^. In this research, the intrinsic cognitive load is highlighted through the task of constructing specific patterns using ”Make ‘N’ Break Extreme” pieces, which are intentionally designed to possess a consistent level of intrinsic complexity.

The second type within CLT is known as *”Extraneous Cognitive Load”*. This arises from the manner in which instructions are presented and the design of the instructional system itself. This type of load, which often results from less effective instructional designs, should preferably be reduced since it has the potential to improperly complicate the learning processes. Fortunately, instructors can manage extraneous cognitive load through careful planning and execution, thereby optimizing instructional delivery to reduce or eliminate its impact^5,13^. In our study, we have applied this concept by incorporating two different instructional methods: visual and code-based to examine their respective influences on cognitive load and performance. The last type defined by CLT is referred to as *”Germane Cognitive Load”*. This concept relates to the cognitive processes that motivate workers to engage actively and exert effort in the learning process. This type of load is crucial for facilitating knowledge acquisition^5,13^. However, in our experimental design, we did not specifically address Germane Cognitive Load as our focus was primarily on examining the effects of work instructions (Extraneous Cognitive Load) while controlling the other types of cognitive load.

In this study, we assess cognitive load both subjectively and objectively. Subjective measures are obtained using both the NASA_TLX^14^ and the short form of the DSSQ^15^, which together provide a comprehensive assessment of multidimensional cognitive workload and dynamic stress states. Short DSSQ focuses on three key psychological states: *engagement*, *distress*, and *worry*. Task *engagement* refers to the individual’s energy level, personal concentration, and task motivation, indicating how strongly someone applies themselves toward achieving goals. Low task engagement is characterized by low energy, reduced motivation, and easy distraction, often manifesting as fatigue. *Distress*, on the other hand, is associated with negative emotional states; it reflects an overload of processing capacity that leads to feelings of lost control and reduced capability. Finally, *worry* involves negative self-assessments and intrusive thoughts that distract from task performance by shifting focus to the personal relevance of the task^16^. Objective cognitive load assessment is evaluated through multiple variables, including physiological indicators: GSR and Heart Rate Variability (HRV) derived from recorded PPG data, hand-motion acceleration, and performance measures like the number of task repetitions, task completion times, and assembly precision.

### Worker performance

In evaluating the effectiveness of work instructions in industrial environments, the performance of workers emerges as a crucial metric. It provides tangible evidence of how well instructions support task execution. This study focuses on several key performance metrics to assess the effectiveness of different instructional methods. One of the primary indicators of effective work instructions is *Task Completion Time (TCT)*. It measures the amount of time required for workers to finish a given task. Successfully accomplishing the task within the designated timeframe, or even earlier, could indicate that the instructions are clear and promote efficient comprehension and implementation. Conversely, prolonged completion times could potentially signify cognitive overload or confusion^17^.

Moreover, evaluating the *Number of Task Repetitions (NTR)* experienced by workers across sessions will provide insight into their ability to efficiently execute and repeat the tasks based on the provided instructions. A higher number of task repetitions can indicate more effective work instructions that facilitate quicker familiarity and mastery of tasks^18,19^. We have utilized a video-based assessment as a method to measure the precision of the worker’s assembly process. Specifically, we define precision as the degree of positional accuracy in placing the blocks, which is quantified by tracking the centers of the attached Aruco markers on each piece. The lower the variance or standard deviation of these positions, the higher the precision. This metric is critical for gauging the relationship between task execution quality and NTR under different instructional methods^7,20,21^.

## Related work

The transition toward Industry 4.0 and 5.0 has brought us to the end of Tayloristic industrial production, a system that breaks tasks into small, standardized steps to maximize efficiency. Modern industrial settings are now distinguished by higher complexity and greater flexibility^22^. Manual assembly is not exempt from these transitions through reducing production depth and increasing reliance on suppliers, and small and more diverse batches^22,23^. This shift leads to less predictability and routine for assembly workers. This uncertainty has increased workers’ workloads and put more pressure on designers to design efficient assembly instructions.

One of the suggested scenarios that has received great attention in recent years is the digital management system, which includes digitally designing and delivering work instructions to individuals. A few examples of these digital techniques are extended reality (XR), augmented reality (AR)^11,24,25^, mixed reality (MR)^26^, digital work instruction supported by multiple video streams^27^, visual contents of work instructions (pictures)^4^ and an approach based on gesture recognition for a self-learning digital assistant system^28^. These techniques can help workers complete their tasks with higher productivity and fewer errors by continuously updating information on the current assembly product, including updates on parts, tools, and processes^22^. However, implementing these new technologies can increase cognitive demands^26^. Furthermore, a significant limitation of many studies is their reliance on subjective metrics, such as questionnaires, and basic performance metrics, like task completion time, without incorporating physiological signals to monitor workers’ cognitive load and performance. While some studies have explored objective indicators using physiological signals, they often lack thorough validation of correlating these objective measures with subjective assessments of both cognitive load and worker performance.

Researchers have employed a wide range of physiological signals to assess cognitive load, including skin conductivity (GSR)^29–32^, photoplethysmography (PPG)^33–35^, electrocardiograms (ECG)^36^, electrooculograms (EOG)^37^, electromyograms (EMG)^38,39^, speech signals^40^, electroencephalograms (EEG)^36,41–43^, acceleration^35,36^, eye blinks, gaze, and movements^44–46^, breathing rate^36,38,46^, skin temperature^36,39^, and blood volume pulse^36^. Most of the studies that utilized these physiological markers to monitor workers’ cognitive load have ranged from standard lab tasks like mathematical problems^29^, the Stroop test^33^, IQ tasks^34^, and constructing with LEGO bricks^37^ to more industrially relevant scenarios such as pushing/pulling wagons and sorting tasks^31^.

Within these contexts, GSR is frequently cited for its sensitivity to stress and arousal^47^, whereas HRV has demonstrated distinct responsiveness to both mental and physical demands. For instance, a study by Taelman et al.^48^ using the wavelet transform of HRV found that tasks involving both mental and physical effort showed similar trends in the High Frequency (HF) parameter as purely physical tasks. However, these tasks had Low Frequency (LF) values, similar to those seen in tasks that were only mentally demanding. In contrast, Garde et al.^49^ found that adding mental challenges to a physical task did not significantly impact HRV parameters. Cheng et al. conducted a study on HRV in individuals engaged in cognitive activities under medium and high physical conditions. The study revealed substantial changes in HRV compared to situations without physical load^50,51^. Given that our experiment encompasses a code-based condition expected to impose significant mental effort yet involve fewer repetitive motions, alongside a visual-based condition anticipated to have lower mental demands but increased physical activity, we integrate HRV and GSR as complementary measures to monitor workers’ cognitive load. Additionally, relatively few studies have systematically evaluated work instructions in assembly tasks while concurrently measuring both subjective (questionnaires) and objective (GSR, HRV) markers of cognitive load.

Following the model proposed by Eesee et al.^52^, who recommended a strategy to manage cognitive load by adjusting workers’ surroundings and the nature of the activity or providing supplementary aids, we designed our experiment that keeps intrinsic task complexity constant-through assembling collections with the same number of pieces each time- while manipulating extraneous load through code-based and visual-based instructions. By doing so, we are applying their criterion to explore how task difficulty management influences the extraneous cognitive load on workers.

This approach extends existing research on digital or simplified instruction methods^22,26^ by explicitly contrasting two instructional formats and validating the outcomes with physiological and self-report data. By examining how workers respond differently in terms of mental effort, stress arousal, and operational efficiency, our study clarifies the balance between offering intuitive guidance and avoiding information overload. This integrated perspective addresses a critical gap in understanding how instructional design can optimize both cognitive and performance outcomes in modern, high-mix industrial environments.

## Methodology

Given the gap identified in the literature, we designed a controlled experiment in which participants assembled “Make ‘N’ Break Extreme” blocks using two instructional methods: code-based and visual-based instructions. This protocol was chosen specifically to isolate extraneous load while maintaining consistent intrinsic load across tasks. The present study aims to investigate the impact of work instructions on operator cognitive load and performance within a controlled, assembly-like scenario. The experiment was carried out in the Industry 5.0 laboratory of the University of Pannonia^53^. In the following subsections of the methodology, we detail the participant recruitment, experimental procedure, data collection, and processing methods used to extract the features from the physiological responses and performance outcomes under each instructional approach.

### Participants

This study recruited 30 participants from the academic field, a mix of university students and researchers with different demographic and ethnic backgrounds. Twelve of them were male and eighteen were female, with ages ranging from 19 to 39 years ($M = 24.733$, $SD = 5.252$). Ethical approval for this study was obtained from the Institutional Review Board of the University of Pannonia (Approval number: KEB_MK_FIT_2024_01). All methods were performed in accordance with the relevant guidelines and regulations. All participants provided written informed consent prior to participation. Since both the visual-based and the code-based instructions rely on colors, participants were required to fill out a vision questionnaire to make sure none of them had color blindness. Three of the participants were wearing contact lenses, and 17 of them had glasses. We also asked the participant to fill out an Edinburgh-handedness questionnaire^54^. Three of the participants were left-handed, and none of the participants had limited hand or finger movements.

### Instructional design

The study involved the use of two instructional approaches for two distinct sessions: *Visual-based* instructions for the low cognitive load session and *Code-based* instructions for the high cognitive load session. In the visual-based session, the participants see a series of step-by-step images depicting exactly how each pair of blocks should connect. In other words, each image clearly shows which sides of the pieces should touch, allowing participants to visually align the blocks until they match the illustrated pattern. The visual instructions presented in this context are characterized by their clarity as they provide a straightforward and unambiguous representation of the final goal. This approach aims to minimize the need for interpretive effort from the participants.

**Fig. 1:**
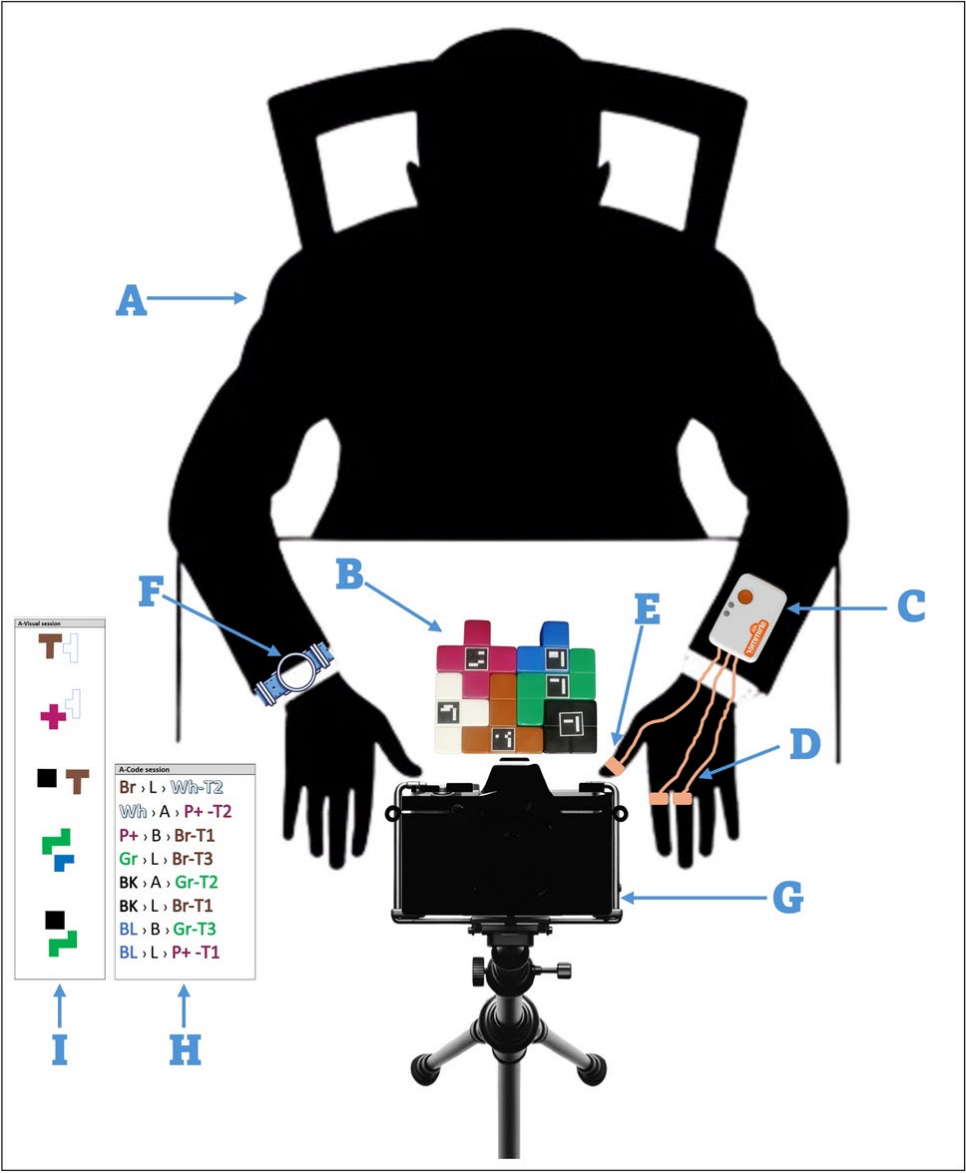
This figure illustrates the comprehensive setup used in our experiment: **Participant (A)**: The participant sits on a chair facing a table where the tasks take place. **Building Blocks (B)**: Displayed on the table are the building blocks used in the experiment, each tagged with an Aruco marker to identify them during the tasks. **Shimmer3 Sensor (C)**: This sensor is attached to the arm of the participant’s non-dominant hand to monitor the physiological signals (GSR and PPG). **GSR Electrodes (D)**: These electrodes are fixed to the proximal phalanx of the index and middle fingers of the non-dominant hand to measure skin conductance. **PPG Electrode (E)**: Positioned on the thumb’s distal phalanx of the non-dominant hand, this electrode monitors the PPG signal. **Metamotion Sensor (F)**: an accelerometer worn on the dominant hand’s wrist; this sensor tracks the participant’s physical motion while engaging in the tasks. **Video Camera (G)**: This camera is mounted on a stand to capture a top-view of the task area. It records the activities during the experiment. **Code-based Instructions (H)**: A sample of code-based instructions provided to participants for task guidance. **Visual-based Instructions (I)**: This is a sample of visual instructions used to direct participants in the experiment.

On the other hand, we utilized a color-based coding system for the assembly instructions to increase the difficulty level in the code-based hard session. A code, usually consisting of the first two letters of its color, references each piece. For example, ‘Re’ signifies the red piece and appears in red text, while ‘Gr’ signifies the green piece and appears in green text. The instructional material provides participants with these codes, which they must use to determine the position and contact points between pieces. The representation of spatial relationships between pieces is denoted by ‘A’ for Above, ‘B’ for Below, ‘L’ for Left of, and ‘R’ for Right of. We denote the degree of contact between two adjacent pieces as ‘T1’ for a single contact region and progressively increase it to ‘T4’ for four contact regions. The codes require participants to translate abstract instructions into the concrete task of assembling the blocks, reflecting a cognitive challenge often encountered in real-life situations where such instructions can be difficult to interpret. Figure 1 shows the setup of the experiment in this study.

### Experiment design and procedure

The experimental setup utilized a customized ”Make ‘N’ Break Extreme Game” construction block set. This set comprises ten distinct blocks, each with a unique color and shape, which are used as the main tools for the work. We attached Aruco markers-square black and white barcode-like stickers-to each block. These stickers enable computer vision algorithms through video-based monitoring to track and verify the precision of the constructions made by the participants.

Each participant completed both visually based and code-based assembly tasks. To counterbalance task difficulty, half of the participants started with the visually-based assembly task, and the other half of the participants started with the code-based assembly task. We created four unique assembly patterns (labeled 1, 2, 3, and 4), each consisting of six distinct blocks. This is to make the tasks more varied and make sure that participants can be properly tested across both instructional approaches. Each participant went through all of them, two for visual-based and two for code-based instructions. To provide a counterbalance and control for the order effect in learning and performance, we further allocated the participants in these two main groups into two subgroups each. Table 1 shows the distributions of the participants for the sessions and the assembly of the patterns.

**Table 1:** Participants distribution and session sequencing in the study of visual and code-Based assembly tasks.

Groups	Sub_Groups	Baseline	Questionnaires	1st Session	Questionnaires	2nd Session	Questionnaires
G1	Sub_G1.1	3 minutes	Pre-DSSQ	Code-based (1 , 2)	Post-DSSQ +NASA_TLX	Visual-based (3 , 4)	Post-DSSQ +NASA_TLX
	Sub_G1.2	3 minutes	Pre-DSSQ	Code-based (3 , 4)	Post-DSSQ +NASA_TLX	Visual-based (1, 2)	Post-DSSQ +NASA_TLX
G2	Sub_G2.1	3 minutes	Pre-DSSQ	Visual-based (1, 2)	Post-DSSQ +NASA_TLX	Code-based (3 , 4)	Post-DSSQ +NASA_TLX
	Sub_G2.2	3 minutes	Pre-DSSQ	Visual-based (3 , 4)	Post-DSSQ +NASA_TLX	Code-based (1 , 2)	Post-DSSQ +NASA_TLX

Before each session, participants engaged in a brief training that corresponds to the specific instructional format, visual, or code-based. The experiment commenced with a three-minute baseline physiological recording. Subsequently, the participants proceeded to complete the pre-DSSQ^15^ to evaluate their stress levels before starting the experiment. Upon finishing the first session, participants completed the post-DSSQ and NASA_TLX questionnaires to evaluate their subjective cognitive load and stress post-task. The process of filling out the post-DSSQ and the NASA_TLX was then repeated at the end of the second session.

Physiological responses during task execution were monitored using the Shimmer3 sensor. Electrodes were attached to the index and middle fingers of the non-dominant hand to record the GSR signal, with its PPG electrode affixed to the earlobe or the thumb for HRV extraction. As physiological signals are sensitive to motion^55^, participants were asked to use their dominant hands only during the assembly task. Furthermore, a Metamotion sensor was employed to track the acceleration of the hand, utilizing its capability as a wearable, wristwatch-like device strapped to the participant’s dominant wrist.

Each session was limited to a total duration of five minutes, during which participants were required to create each specified pattern a minimum of three times for the purpose of learning curve analysis. The duration of the sessions could be longer than five minutes, just in the cases where the participant has not met the minimum number of task repetitions (NTR). Time-stamped data from each session was captured to track progress and performance.

### Data preprocessing

**Fig. 2:**
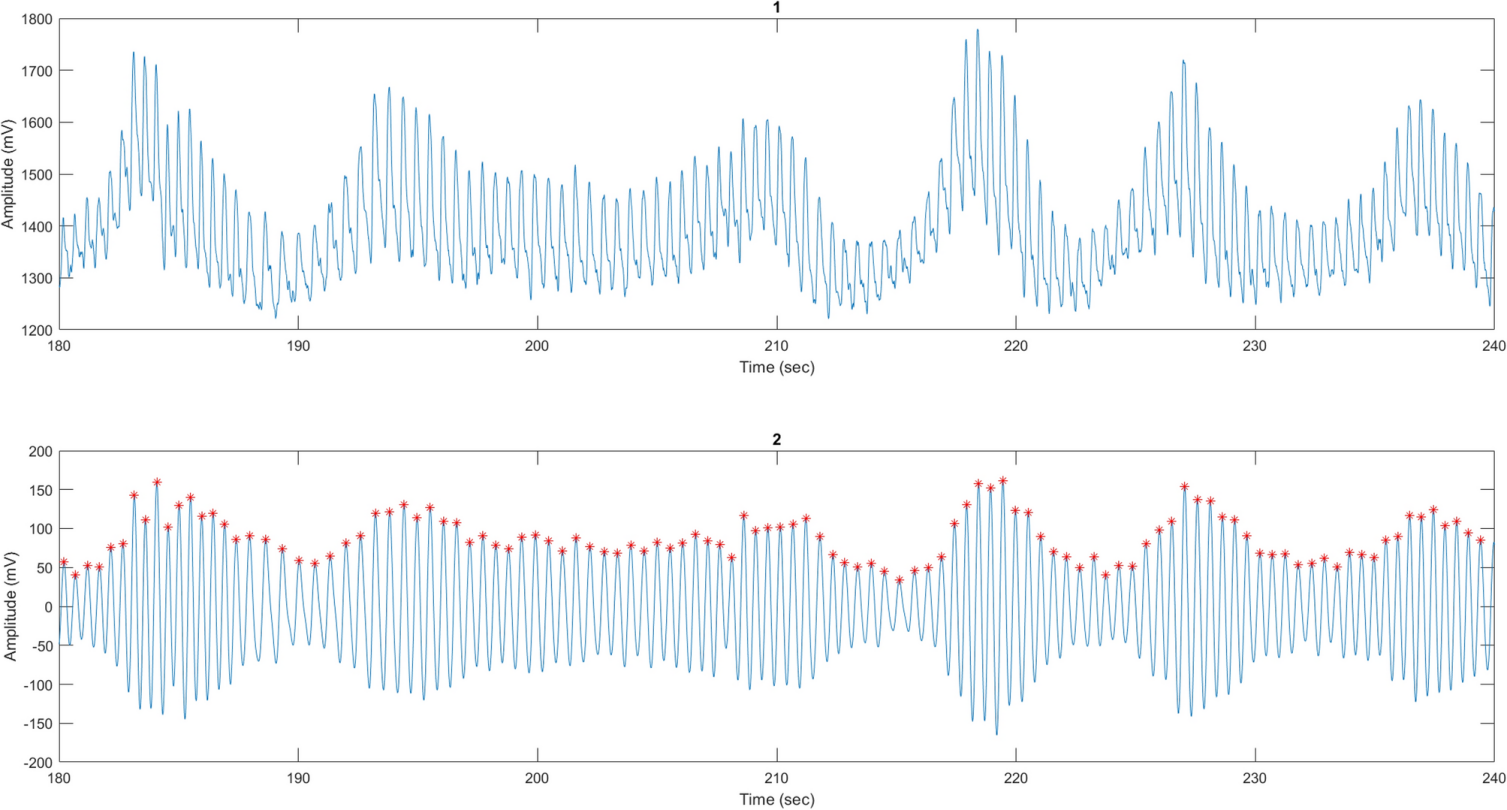
(1) A sample of Raw PPG signal for 60 seconds, (2) The same sample of the PPG signal after being filtered and removing the DC offset and low-frequency trend with its detected peaks.

In this experiment, we set the sampling frequency of the Shimmer3 sensor to 250 Hz to capture the physiological signals GSR and PPG. Low-frequency trend noise accompanies most of the recorded PPG signals, which complicates direct HRV extraction. We started with mean correction by removing the DC level offset to make sure that the signals are oscillating around the zero baseline. Following this step, we implemented the Savitzky-Golay filter to remove the low-frequency trend noise. We then implemented the peak detection technique to extract the distances between the peaks and obtain the HRV. Figure 2 shows a sample of a 60-second PPG signal before and after removing the DC offset and low-frequency trend.

To increase the data size, we applied a 60-second segmentation window to the filtered signals. We extracted HRV signals for each 60-second window by calculating the variation between consecutive detected peaks on the time axis. We extracted 19 features from the HRV signals for each window. The summarized HRV features extracted in this study are presented in Table 2.

**Table 2:** The list of the extracted HRV features and their description.

HRV acronyms	Description
RMSSD	The square root of the average of the squared differences between consecutive intervals:
	$\frac{\sqrt{\sum _{i=1}^{N-1} (RR_{i+1} - RR_i)^2}}{N-1}$, RR is the interval between the peaks^56^.
MEAN	The mean of the RR intervals.
MEDIAN	The median of the RR intervals.
SDRR	Standard Deviation of the RR intervals.
SDSD	Standard deviation of the differences between consecutive RR intervals.
SDRR_RMSSD	Ratio of SDRR to RMSSD.
HR	Heart Rate (beats per minute).
PNN25	Percentage of consecutive RR intervals differing by more than 25 ms.
PNN50	Percentage of consecutive RR intervals differing by more than 50 ms.
SD1	Descriptor of short-term HRV from the Poincaré plot.
SD2	Descriptor of long-term HRV from the Poincaré plot.
KURT_RR	Kurtosis calculated from all RR intervals.
SKEW_RR	Skewness calculated from all RR intervals.
VRL	Power spectrum of the Very low frequency band (0.003 Hz to 0.04 Hz) of the HRV.
LF	Power spectrum of the low frequency band (0.04 Hz to 0.15 Hz) of the HRV.
HF	Power spectrum of the high frequency band (0.15 Hz to 0.4 Hz) of the HRV.
TP	Total power spectrum of the HRV.
LF_HF	The ratio of the LF to HF.
HF_LF	The ratio of HF to LF.

The GSR signal (also known as the skin conductance SC) is formed by superimposing the phasic SC, also called the skin conductance response (SCR), on the tonic SC (also called the skin conductance level SCL), which is slowly changing^57^. This concept dictates that $SC = SC_{{\rm tonic}} + SC_{{\rm phasic}}$. Monitoring SCR is a simple way to detect sympathetic activity in response to an event^58^. Based on these facts, using the SC as a monitor for the change in sympathetic activity requires a technique to separate the signal into its phasic and tonic levels. We have utilized the Matlab-based Ledalab software V3.4.9, which uses a standard deconvolution algorithm to separate the SC into its two components^58^. Before starting the separation process, we applied a built-in adaptive smoothing filter to the signals to remove their noise. We initiated the separation process by applying continuous decomposition analysis (CDA).

**Fig. 3:**
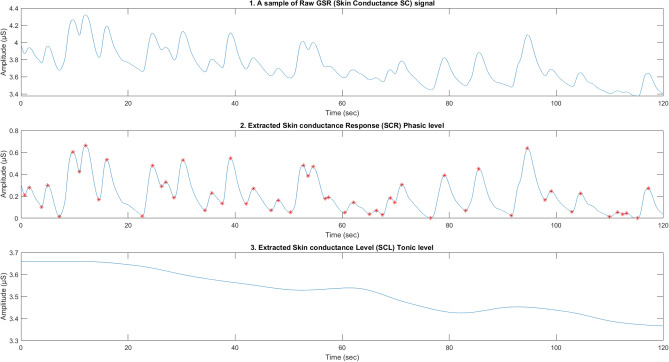
(1) A sample of the recorded GSR for 120 seconds; (2) The extracted SCR through continuous decomposition analysis (CDA) with extracted peaks and bottoms that are utilized for feature extraction; and (3) The extracted SCL through the same CDA analysis.

We also employed a 60-second segmentation on the extracted signals, ensuring consistency in sample size with previous HRV measurements. From the GSR and its two components, SCR and tonic SC, we extracted 9 features. Personal differences in skin conductivities influenced the amplitudes of both SC components (SCR and tonic SC). As our study focuses on the effects of work instructions, we manually checked the processing steps during feature extraction. This approach enhanced the robustness of our methods, helping us avoid irrelevant details and preserve critical and subtle features. Figure 3 shows a sample of 2 minutes of GSR recording with its two components (SCR and SCL). Table 3 shows the list of the extracted GSR features.

**Table 3:** The list of the extracted GSR features and their description.

GSR acronyms	Description
AreaSCR	Total area under the SCR curve
AreaGSR	Total area under the SC curve
No_Peakes	Number of the detected peaks in the SCR
AvgRiseTime	Average of the rising time of the peaks
AvgDecayTime	Average of the decaying time of the peaks
Entropy	Measured entropy of the SC signal
STDGSRdata	Standard deviation of the SC
STDSCRdata	Standard deviation of the SCR
BandPower	Summation of the power spectrum of the SCR

For acceleration data recorded from the sensor on the dominant hand’s wrist, we captured three data axes: *X*, *Y*, and *Z*. Consistent with previous physiological data, we applied 60-second segmentation on the acceleration signals. We calculated the resultant of these three axes and extracted six features from each axis, resulting in 24 features. These features included mean, median, standard deviation, minimum, and maximum. All the signal processing steps, including filtration, feature extraction, and segmentation for GSR, HRV, and acceleration data, were conducted using MATLAB 2024b^59^.

Finally, the precision of each constructed pattern was evaluated through a customized algorithm. This algorithm processes video-captured images and analyzes the placement and orientation of each piece via Aruco markers. It calculates the Euclidean distance between the centers of the markers in the constructed pattern and compares it against a reference, whereby variances are determined as a measure of standard deviation. A higher value of the standard deviation indicates lower assembling precision, while a lower value suggests higher precision. This analysis was implemented using Python within the Spyder 5 environment^60^

## Results

### Subjective data analyses

In this subsection, we analyze the subjective data collected from participants during the three sessions of the experiment. We utilized two questionnaires, the NASA_TLX and the short version of the DSSQ. These questionnaires capture the perceptions of the participants after each session of the experiment.

#### NASA_TLX questionnaire

**Fig. 4:**
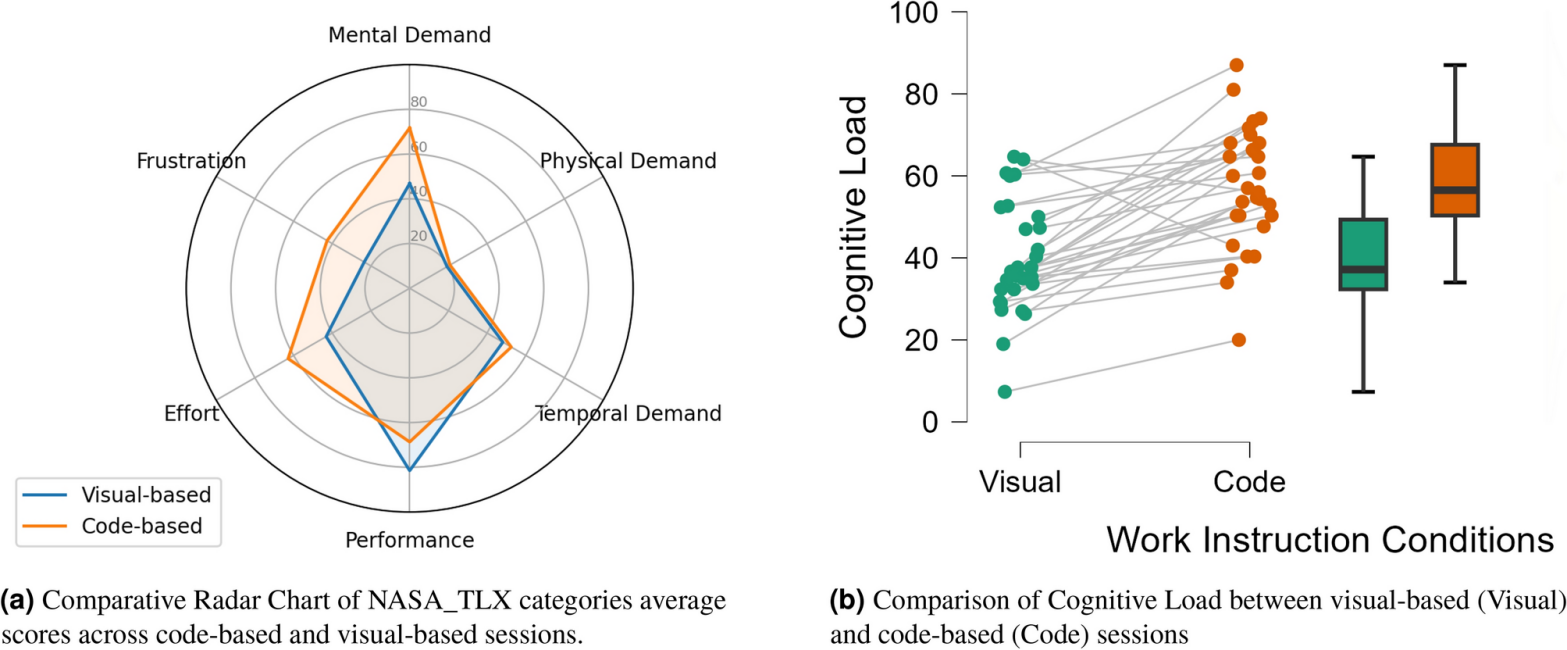
Comparative analysis of subjective Cognitive Load and NASA_TLX dimensions across visual-based and code-based sessions.

The NASA_TLX expresses six categories as percentages: mental demand, physical demand, temporal demand, performance, effort, and frustration. Figure 4a is a radar chart to visually compare these categories between the code-based and visual-based work instructions sessions. It shows that the code-based instructions induced higher levels of mental demand, frustration, and effort compared to the visual-based instructions. The statistical paired t-test confirmed significant differences between them, with $p-values < 0.001$ and effect sizes of −1.522 for mental demand, −0.788 for frustration, and −0.913 for effort. These findings indicate that code-based instructions were more mentally demanding and frustrating, requiring more effort to decipher than visual-based instructions. Additionally, the results showed slightly higher levels of both physical and temporal demand for the code-based instructions compared to the visual-based instructions. However, these differences were not statistically significant. The $p-value$ for physical demand was 0.775 with an effect size of −0.082, and for temporal demand, the $p-value$ was 0.339 with an effect size of −0.177. This suggests that participants did not feel rushed by time constraints, but they were more challenged by aspects related to their limited working memory.

Finally, the nature of the NASA_TLX scale interprets the “performance” dimension in the opposite direction of the other five categories, yet assigns its weight in the same direction as the others. This means that a higher perceived performance results in a lower NASA_TLX score, contributing to a lower overall cognitive load. For visual clarity in our radar chart, we assigned the performance weight in the reverse direction to the other categories to reflect each participant’s self-perceived performance. According to the radar chart and the paired t-test, participants reported higher perceived performance with the visual-based instruction compared to the code-based instruction, with a significant difference ($p-value < 0.001$ and Effect Size = −0.852).

The cognitive loads (CLs) for the visual-based (Visual) and code-based (Code) sessions were compared in Fig. 4b. These CLs were calculated from the NASA_TLX categories. The figure presented a combination of individual data points (personal CLs) with paired lines and box plots. The lines connecting the dots across the two sessions indicate the shift in CL for each participant from ”Visual” to ”Code”, highlighting a general increase in CLs in the code-based session.

The box plot shows the distribution of CLs in both sessions, with a higher median and wider interquartile range in the code-based session. This suggests more variability and a higher overall CL. The mean values of the two sessions align with these box plots, where the visual-based session gave a *Mean* of 39.84 with an *SD* of 13.74 compared to the code-based session, which gave a *Mean* of 57.24 with an *SD* of 14.71. An analysis using a paired t-test supported these observations. It showed that CL increased significantly from the visual-based session to the code-based session ($P < 0.001$), with an effect size of −1.182. This indicates that code-based instructions, compared to visual-based ones, place a significantly higher cognitive demand on participants. The statistical results for the comparisons of cognitive load and each NASA_TLX dimension between the visual-based and code-based sessions are summarized in Table 4.

**Table 4:** Statistical comparison of NASA_TLX Cognitive Load and its dimensions between visual-based and code-based instruction sessions.

Measure 1 Visual-session	Measure 2 Code-session	Shapiro-Wilk *p*-value	Test	Z	Effect size	t-test *p*-value
Cognitive load	Cognitive load	0.208	Student	N/A	−1.182	<0.001
Mental demand	Mental demand	0.150	Student	N/A	−1.522	<0.001
Physical demand	Physical demand	<0.001	Wilcoxon signed-rank	−0.305	−0.082	0.775
Temporal demand	Temporal demand	0.575	Student	N/A	−0.177	0.339
Performance	Performance	0.114	Student	N/A	−0.852	<0.001
Effort	Effort	0.942	Student	N/A	−0.913	<0.001
Frustration	Frustration	0.125	Student	N/A	0.788	<0.001

#### Short DSSQ questionnaire

**Fig. 5:**
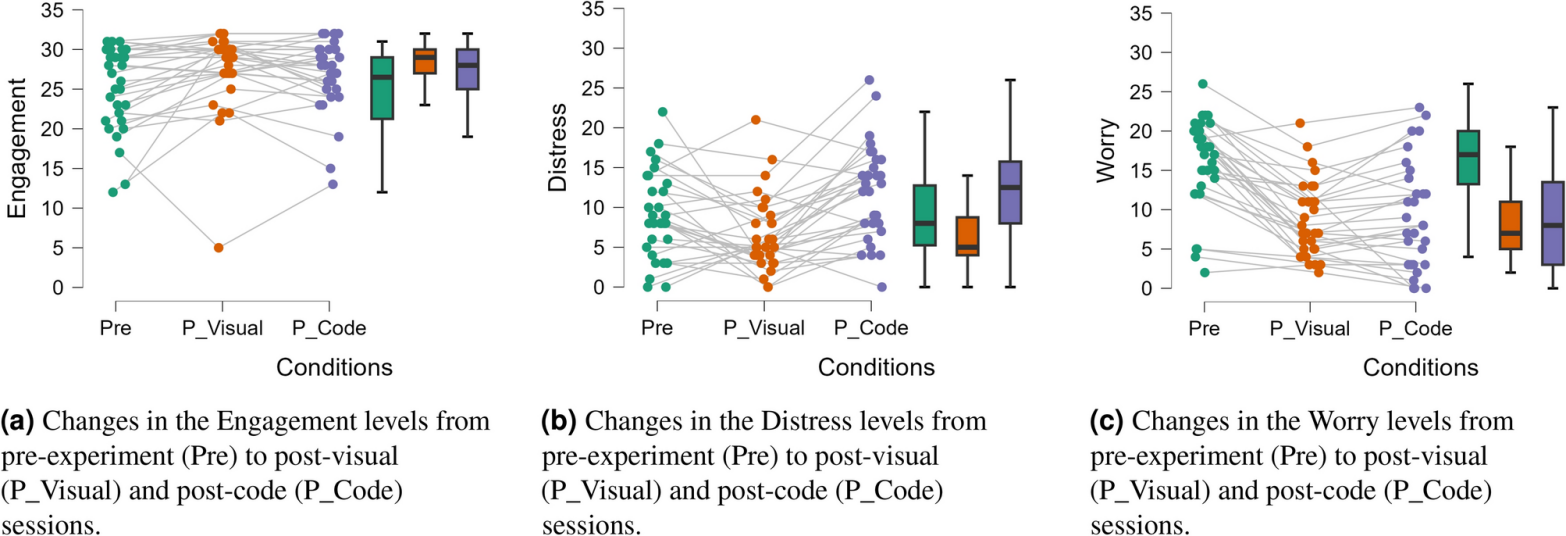
Engagement, distress, and worry psychological states derived from the short DSSQ across three different conditions.

The second questionnaire we utilized in this experiment was the short version of DSSQ. Participants in this study completed the DSSQ three times under the following conditions: pre-experiment, post-visual-based session, and post-code-based session. The scores for each of the three psychological states were in the range of $(0-32)$. Figure 5a displays the engagement scores of each individual, connecting them across the sessions to illustrate changes in engagement for each participant. Boxplots summarize the distribution of scores within each condition, providing a clear visual comparison.

The descriptive statistics for the engagement scores revealed variations across the three sessions. Prior to the experiment (Pre), the *Mean* engagement score was 25.06, with an *SD* of 5.33. Following the visual-based session (P_Visual), the *Mean* engagement score increased to 27.43, accompanied by a *SD* of 5.21. Following the code-based session (P_Code), the *Mean* engagement score decreased slightly to 26.83, with a *SD* of 4.75.

To check if study sessions had a significant effect on task engagement, we decided to implement the Repeated Measures RM ANOVA. To check the assumption of sphericity, we applied Mauchly’s test, which revealed a violation of assumptions with a $p-value$ of 0.043. Therefore, we applied the Huynh-Feldt correction to account for this violation. The corrected repeated measures ANOVA identified significant differences in engagement scores across the conditions, with a significant $p-value$ of 0.012.

Following the main RM ANOVA test, we also applied the Post-Hoc analysis. We observed a significant increase in engagement from the pre-experiment to the post-visual-based session, as evidenced by a significant $p-value$ of 0.041 and an effect size of −0.464. Although the increase in engagement from the pre-experiment to the post-code-based session had a *p*-value of 0.059 with effect size of −0.346, it did not meet the conventional significance threshold of 0.05. Finally, a *p*-value of 0.323 and an effect size of 0.118 indicated no significant differences between the post-visual-based and post-code-based sessions. This suggests that both types of instructions managed to sustain similar levels of engagement (see Fig. 5a). These results indicate that while both instructional methods effectively boosted engagement compared to the baseline, the visual-based instructions proved particularly effective, as reflected in higher mean engagement scores.

We further looked into the distress scores of participants across three different sessions. There was a notable variation in these scores. Initially, before the experiment (Pre), the *Mean* distress score was 9.00, with an *SD* of 5.52. After the visual-based session (P_Visual), the distress scores decreased to a *Mean* of 6.56 with an *SD* of 4.76. However, following the code-based session (P_Code), the *Mean* distress score increased to 11.83, with an *SD* of 6.02 (see Fig. 5b).

To figure out if these changes in distress scores were statistically significant, we applied the RM ANOVA test, followed by the Post-Hoc tests. As before, for robust analysis, we applied Mauchly’s test to check for sphericity. The test results showed no violations ($p-value = 0.792$), which meant that we could use a standard repeated measures ANOVA without any adjustments. The ANOVA results indicated that there were indeed significant differences in distress scores across the conditions, with a highly significant $p-value$ of less than 0.001.

In the Post-Hoc tests of the ANOVA results, we took a closer look at the changes in distress scores between the sessions. We found a significant decrease in distress from the pre-experiment to the post-visual-based session, with a $p-value$ of 0.030 and an effect size of 0.446. Furthermore, the transition from the pre-experiment to the post-code-based session revealed a similar significant increase in distress, with a $p-value$ of 0.030 and an effect size of −0.519. Most notably, the transition from the post-visual-based session to the post-code-based session marked a substantial increase in distress levels, with a $p-value < 0.001$ and a high effect size of −0.964.

Distress, which is linked to negative emotional states, was initially high, as demonstrated by the pre-experiment mean scores, indicating significant initial stress among participants. However, after using visual instructions, there was a noticeable drop in distress levels, indicating a sense of relief. In contrast, the distress levels increased sharply after the code-based sessions, suggesting that these instructions significantly heightened negative emotional states which is related to the overload of processing capacity. This pattern demonstrates the substantial impact that different instructional designs can have on participants’ psychological stress. The marked differences between the visual and code-based sessions highlight the need to carefully consider the type of instructional material used and its potential psychological effects on learners. (Refer to Fig. 5b for a visual representation of these results.)

Next, we looked into the final psychological state, Worry. The worry scores changed notably across sessions. At the pre-experiment (Pre), the *Mean* worry score was quite high, at 15.86, with an $SD = 5.85$. After the visual-based session (P_Visual), this score significantly dropped to 8.60 ($SD = 4.86$), showing a large reduction in worry. However, after the code-based session (P_Code), the *Mean* worry score increased slightly to 9.23, with an $SD = 6.88$ (see Fig. 5c). To validate these observations, we first looked at the assumption of sphericity using Mauchly’s test. It showed a violation ($p-value = 0.037$). Consequently, we applied the Huynh-Feldt correction before proceeding with a RM ANOVA. This analysis confirmed that there were significant differences in worry scores across the sessions, with a highly significant $p-value < 0.001$.

In the Post-Hoc tests of the ANOVA, worry greatly decreased from the pre-experiment to the post-visual-based session, with a $p-value < 0.001$ and a large effect size of 1.226. Similarly, worry significantly decreased from the pre-experiment to the post-code-based session, with a $p-value < 0.001$ and an effect size of 1.120. However, the worry scores did not significantly change from the post-visual-based to the post-code-based session ($p-value = 0.397$, effect size = −0.107). This suggests that the code-based session did not negatively affect the initial reduction in worry. These statistics for the DSSQ’s three variables-engagement, distress, and worry-along with the Post-Hoc analysis, are presented in Table 5.

**Table 5:** Statistical analyses of DSSQ variables (Engagement, Distress, and Worry) with Post-Hoc comparisons.

DSSQ states	Sphericity test Mauchly *p*-value	Sphericity Correction	RM ANOVA *p*-value	ANOVA Post-Hoc		
				Cases	*p*-value	Effect size
Engagement	0.043	Huynh-Feldt	0.012	Pre vs. P_Visual	0.041	−0.464
				Pre vs. P_Code	0.059	−0.346
				P_Visual vs. P_Code	0.323	0.118
Distress	0.792	None	<0.001	Pre vs. P_Visual	0.030	0.446
				Pre vs. P_Code	0.030	−0.519
				P_Visual vs. P_Code	<0.001	−0.964
Worry	0.037	Huynh-Feldt	<0.001	Pre vs. P_Visual	<0.001	1.226
				Pre vs. P_Code	<0.001	1.120
				P_Visual vs. P_Code	0.397	−0.107

Pre: Pre-experiment, P_Visual: Post Visual-based session, and P_Code: Post Code-based session.

These findings show that the way instructions are designed can greatly affect worry, which is linked to negative self-assessments. The large decrease in worry scores after the visual-based session suggests that this method can effectively reduce worry, helping participants focus better and feel more comfortable. On the other hand, the slight increase in worry after the code-based session, although not significant compared to the visual session, shows that certain instructional methods might make anxiety worse under specific conditions. The visual representation of these results is shown in Fig. 5c. All statistical analyses, including t-tests, RM ANOVA, and Post-Hoc comparisons, were conducted using JASP statistical software (version 0.19.2)^61^.

### Objective data analyses

In this subsection, we present the results of the analyses based on the captured objective data. Starting with the recorded physiological data, we extracted 19 HRV features, and nine GSR features listed before respectively in Tables 2 and 3. Feeding all of these physiological features for classification purposes or even statistical analyses can lead to poor accuracy and precision because some of these features could be highly correlated while others may not show a high contribution to predicting the target. Based on this criterion, it is inevitable to implement the feature selection technique prior to classification processes. Wrapper methods are the most effective for feature selection, according to Rezaei and Jabbari^62^. We implemented a feature selection technique that belongs to the wrapper methods: backward elimination. This technique is based on employing the entire set of features in the first step and gradually iterating and removing the features. Each iteration removes the feature that contributes the least to the target. This process continues as long as the model improves with feature removal.

Due to the nature of our experiment design, the data from the three sessions is not equally sized. Repeated analyses with varying sizes contravene standard statistical analyses such as the ANOVA and paired t-test. However, we can use logistic regression analysis for this purpose. The rationale for utilizing logistic regression lies in its ability to provide classification properties, in addition to displaying the contribution of each feature to the target along with its *p*-value. We have utilized SPSS statistical software for this purpose. We fed the 29 extracted features into the model, using the backward elimination method to iterate over them and select the most significant ones. Following the designed sessions of this experiment (refer to Table 1), we will compare the whole three sessions and each session with the other two sessions separately, similar to the Post-Hoc tests in the subjective analyses.

We aim to provide a comprehensive overview of the impact of the type of work instruction on physiological features. We used a multinomial logistic regression model, setting the baseline session as the reference category for the visual and code-based sessions. This means that the features of both sessions of work instructions will be compared to the baseline session. The fitness of the model was assessed using the Chi-Square test, which revealed a significant improvement over the null model with Chi-Square = 270.503, $d = 30$, and a $p-value < 0.001.$

The backward elimination method removed 14 features and selected the top 15 contributing features, resulting in the optimal model classification parameters. Table 6 presents a list of the selected features, as well as their coefficient magnitudes and *p*-values. We also implemented three binary logistic regression models to compare each session with the others and see which features had contributed significantly to the target. We once again assessed the models’ fitness using the Chi-Square test. The results revealed a significant improvement in the models compared to the null models without predictors. The visual-based vs. baseline model has shown Chi-Square = 140.358, $d = 12$, and a $p-value < 0.001$, the code-based vs. baseline model has shown Chi-Square = 176.691, $d = 13$, and a $p-value < 0.001$, and the code-based vs. visual-based model has shown Chi-Square = 91.401, $d = 14$, and a $p-value < 0.001$. Table 7 presents the results of the selected features with their coefficient magnitudes and significance evaluation parameter, *p*-values. Negative coefficients suggest that as the predictor (a specific feature) increases, the likelihood of the outcome being in the respective condition (target) decreases compared to the reference category.

By comparing the three conditions in our study, we calculated and presented the average of the models’ performance parameters-*accuracy*, *precision*, and *recall*-in Table 8. We used the following abbreviations for each condition, B: Baseline (Pre-experiment) session, V: Visual-based instruction session, and C: Code-based instruction session. The high-performance classification metrics (B-C, B-V) showed that the models found a clear boundary between the baseline condition (Pre-experiment) and both the visual-based and code-based conditions. This pattern aligns with the trends observed in the ANOVA and Post-Hoc tests of the DSSQ psychological states: engagement, distress, and worry. Although all selected features of condition 3 (Code based vs. Visual based) in Table 7 demonstrated significant differences, model V-C in Table 8 exhibited the lowest performance metrics when compared to the other binary classifier models. This is somewhat consistent with the previous statistical tests that compare these two conditions within the subjective DSSQ states.

To analyze participants’ performance, we analyzed three key metrics: the Number of Task Repetitions (NTR), Task Completion Time (TCT) (a minimum of five minutes), and the precision of the assembly process. We represented this precision by the average of the standard deviation (SD) of Euclidean distances between the centers of the building pieces, derived from video-captured images; the lower the SD value, the better the assembly process. We calibrated the camera setup and averaged the trials within each instruction session to minimize potential algorithmic inaccuracies. We conducted Shapiro-Wilk tests to assess the normality of the data, followed by paired t-tests to evaluate differences between sessions.

**Fig. 6:**
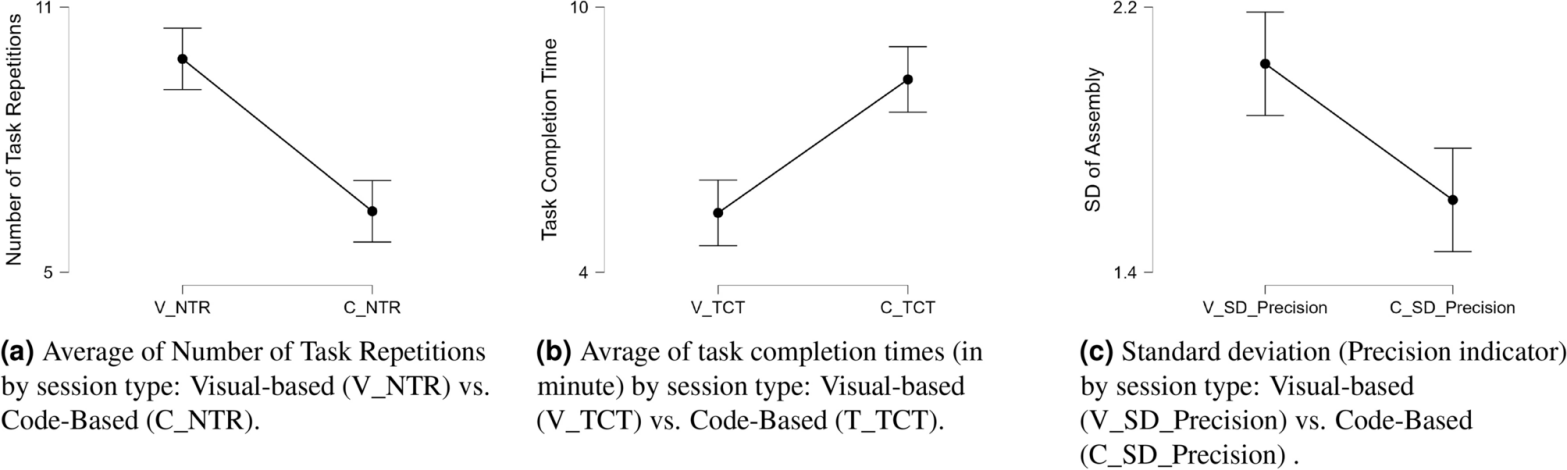
Comparative analysis of task performance across visual and code-based instruction sessions.

Table 9 shows the main parameters extracted from these tests. We observed significant differences in the three parameters between the two sessions, with *p*-values $< 0.001$. In Fig. 6, we present the descriptive plots of these three parameters as means with their confidence intervals 95%. Figure 6a presents the means of the NTR during the two sessions of the work instructions. In the code-based session, most of the participants found themselves stuck at the minimum number of iterations ($Mean = 6.379$), whereas they showed a greater capability to repeat the task in the visual-based session ($Mean = 9.828$).

**Table 6:** Estimated parameters for selected features using multinomial logistic regression with baseline session as reference.

Condition	Features	B	*p*-value	Condition	B	*p*-value
Visual-based	AreaGSR	0.007	< 0.001	Code-based	0.007	< 0.001
	NoPeakes	0.295	< 0.001		0.133	0.049
	avgRiseTime	0.238	0.11		0.338	0.017
	avgDecayTime	− 0.0001	0.957		−0.228	0.006
	STDGSRdata	−17.15	< 0.001		−13.008	< 0.001
	STDSCRdata	29.078	< 0.001		27.274	< 0.001
	spectralEnergy	−0.215	< 0.001		−0.389	< 0.001
	MEAN_RR	−0.018	0.006		−0.028	< 0.001
	SDRR	−0.147	< 0.001		−0.015	0.634
	SDSD	0.139	< 0.001		0.049	0.114
	SDRR_RMSSD	2.708	0.028		0.068	0.953
	HR	−0.218	0.01		−0.344	< 0.001
	pNN25	−0.019	0.301		−0.059	< 0.001
	LF_HF	−0.004	0.034		−0.006	0.001
	HF_LF	0.544	0.784		1.809	0.362

The reference category is: Baseline session. B: Coefficient Magnitudes

**Table 7:** Estimated parameters for selected features using three binary logistic regression models.

Condition 1	Features	B	*p*-value	Condition 2	Features	B	*p*-value	Condition 3	Features	B	*p*-value
Visual-based vs Baseline	AreaGSR	0.006	0.003	Code-based vs Baseline	AreaSCR	0.465	< 0.001	Code-based vs Visual-based	NoPeakes	−0.158	< 0.001
	NoPeakes	0.164	0.005		NoPeakes	0.165	0.010		avgDecayTime	−0.200	0.032
	STDGSRdata	−13.921	0.001		STDGSRdata	−9.952	0.006		STDGSRdata	8.201	0.001
	STDSCRdata	22.950	< 0.001		spectralEnergy	−3.746	0.005		STDSCRdata	−8.528	0.016
	spectralEnergy	−0.177	0.006		bandPower	7.032	0.018		spectralEnergy	−1.177	0.016
	RMSSD	0.091	< 0.001		MEAN_RR	−0.031	< 0.001		bandPower	2.152	0.036
	MEDIAN_RR	−0.011	0.027		SDSD	0.092	< 0.001		MEAN_RR	−0.078	0.008
	SDRR	−0.054	0.003		HR	−0.753	0.003		SDSD	−0.056	< 0.001
	HR	−0.129	0.025		pNN25	−0.047	0.034		SDRR_RMSSD	−2.664	< 0.001
	pNN25	−0.048	0.023		pNN50	−0.095	0.020		HR	−0.546	0.005
	KURT_RR	−0.072	0.087		KURT_RR	−0.093	0.007		pNN50	−0.051	0.003
	LF_HF	−0.003	0.117		VLF	0.000	0.048		SD2	0.076	< 0.001
					LF_HF	−0.005	0.029		LF	0.000	0.015
									HF_LF	1.543	0.002

Baseline is the reference category for Conditions 1, and 2, while Visual-based is the reference in the Condition 3. B: Coefficient Magnitudes

**Table 8:** Average of performance metrics of logistic regression models under various conditions based on the physiological features (GSR and HRV).

Classifier	Conditions	Accuracy	Precision	Recall
Multinomial Logistic Regression	B–V–C	78.04	67.79	63.12
Binary Logistic Regression	B–V	83.88	82.99	82.2
	**B–C**	**90.42**	**89.97**	**85**
	V–C	75.51	74.92	71.95

B: Baseline (Pre-experiment), V: Visual-based session, and C: Code-based session

Despite the higher number of repetitions in the visual-based session, the majority of participants did not exceed the allocated time for this session and showed a mean of 5.342 minutes compared to 8.363 minutes in the code-based session (refer to Fig. 6b). These results are aligned with the subjective results from NASA_TLX, where participants showed a significant increase in the cognitive load from visual-based instructions to code-based instructions. Figure 6c, however, showed intriguing results with lower SD values for code-based instructions (which means better precision) compared to visual-based instructions. This does not align with the subjective results of the NASA_TLX performance category, where participants evaluated themselves as having better visual instructions performance.

**Table 9:** Comparison of participant performance metrics between Visual- and Code-based sessions using paired t-tests.

Measure 1	Measure 2	Shapiro-Wilk *p*-value	Test	Z	Effect size	t-test *p*-value
V_NTR	C_NTR	0.027	Wilcoxon signed-rank	4.286	1.00	< 0.001
V_TCT	C_TCT	0.018	Wilcoxon signed-rank	−4.573	−0.972	< 0.001
V_SD_Precision	C_SD_Precision	0.3	Student	N/A	0.709	< 0.001

V_NTR and C_NTR are the Number of Task Repetitions in the visual- and code-based sessions respectively, V_TCT and C_TCT are the Task Completion Time (in minute) of the visual- and code-based sessions respectively, and V_SD_Precision and C_SD_Precision are the SDs that reflect the precision of the assembly process for the visual- and code-based sessions respectively

The higher number of task repetitions NTR and lower task completion time TCT in the visual-based session indicate that participants made more hand movements in this session compared to the code-based session. Table 10 shows the t-test analyses of the hand movements in the three coordinates (X, Y, Z) during two work instructions. The *p*-values from these analyses (< 0.05) indicate significant differences in hand movement across the three coordinates during the visual-based session compared to the code-based session, highlighting variations in NTR and TCT between the two sessions.

The accelerometer data in the three coordinates provided 24 features as already explained in the Data Preprocessing subsection. Combining these features with 29 features that were previously extracted from the physiological signals (GSR and PPG) will establish a clear boundary between the two instruction sessions. Feeding these 53 features into the binary logistic regression model with the backward elimination method has produced promising results. We again assessed the fitness of the model using the Chi-square test, revealing a significant improvement over the null model with Chi-square = 368.234, $d = 27$, and a $p-value < 0.001.$ The model showed excellent performance metrics with average $accuracy = 92.91$, $precision = 92.67$, and $recall = 92.35$. These values outperform the model performance in Table 8 V-C condition. All statistical analyses related to the logistic regression modeling were conducted using IBM SPSS Statistics software (version 29)^63^.

**Table 10:** Results of t-test analyses comparing the mean accelerometer values across the three coordinates (X, Y, Z) during two work instruction sessions: visual-based and code-based.

Visual-based	Code-based	Test	Z	*p*-value
meanX	meanX	Wilcoxon signed-rank	2.411	0.016
meanY	meanY	Student	N/A	0.002
meanZ	meanZ	Wilcoxon signed-rank	2.038	0.042

meanX, meanY, and meanZ are the mean values of the accelerometer data at the three coordinates X, Y, and Z respectively

## Discussion

This study investigated the impact of work instruction methods on the human cognitive load and their operational efficiency. In a controlled, assembly-like scenario inspired by industrial tasks, the study used two work instructions-visual-based and code-based-and a range of subjective and objective assessment methods. The study also examined the alignment between subjective and objective evaluation methods, in order to enhance the accuracy of conclusions by providing context for physiological responses and validating our experimental conditions. The findings revealed that code-based instructions imposed a higher subjective cognitive load on participants compared to visual-based instructions. This aligns with Cognitive Load Theory (CLT), which posits that extraneous cognitive load-stemming from the way information is presented-can hinder learning and performance^13^.

The results are also consistent with previous studies indicating that visual aids can enhance comprehension and reduce cognitive load in assembly tasks. For instance, Li et al. (2018) found that supporting the work instructions with pictures can reduce the cognitive load and improve task performance compared to the traditional text instructional methods^4^. Similarly, our study suggests that visual-based instructions lead to faster task completion and higher task repetition rates. This is likely due to the reduced mental effort required to interpret the instructions, as visual-based instructions are less abstract and easier to interpret than code-based instructions. Furthermore, Vanneste et al. (2024) demonstrated that augmented reality (AR) visual instructions led to lower assembly times and a lower perceived physical effort compared to traditional methods^11^. This supports the idea that technologically advanced visual aids can further enhance the effectiveness of work instructions, which aligns with our findings on the superiority of visual-based instructions in most cases.

Taking each of the six categories in the NASA_TLX and comparing them between the two instructional sessions has produced profound results. The t-test analyses of each pair of the six categories within the NASA_TLX have shown a significant increase in mental demand, frustration, and effort in the code-based session. While there was a slight increase in physical demand in the code-based session, there was no significant increase. Conversely, the t-test analyses of hand movements in the X, Y, and Z coordinates, as well as the NTR, indicated higher means and significant differences in the visual-based session compared to the code-based session. As the hand movements were not exertive, participants focused on their goal of repeating the task during the visual-based session, where a higher repetition rate was intended to lead to better outcomes. Consequently, they did not perceive the task as physically demanding when filling out the Physical Demand category of NASA_TLX.

However, body movement significantly impacts physiological signals due to the alterations in autonomic sympathetic arousal resulting from increased energy expenditure^64^. Subjectively, the code-based instructions were more cognitively demanding. We also expected these instructions to influence the objective physiological data. On the other hand, although visual-based instructions posed subjectively lower cognitive demands, their straightforward nature objectively led to a higher number of hand movements. We expect the higher hand movements to impact the objective physiological data. These were clearly reflected in the performance metrics of the logistic regression models in Table 8. Classifying the code-based instruction session from the baseline session yielded the highest performance metrics, with the visual-based instruction session from the baseline session following closely behind. Both cognitively demanding tasks and tasks involving body movements significantly influence the physiological signals, justifying this. Simultaneously, when we attempted to classify the code visual-based sessions, the logistic regression models displayed relatively low-performance metrics because both tasks were objectively influencing the physiological signals.

The low-performance metrics for classifying the two sessions based on physiological data do not necessarily indicate a lack of alignment between the subjective and objective data metrics. However, they do imply that differentiating operators’ conditions using the objective physiological data may not be entirely reliable, especially in scenarios combining cognitive and physical tasks. On the other hand, supporting the features extracted from the physiological signals (GSR and HRV) with the features extracted from the accelerometer has provided a clear boundary between the two instruction sessions. This is due to the higher levels of hand movements in the visual-based session. This supports the use of these kinds of signals in conjunction with other objective data to support operator condition analyses.

This study also used performance as a metric. We informed the participants about the criteria for evaluating their performance prior to the experiment. We analyzed this metric in two ways: subjectively using the NASA_TLX, and objectively using the parameters in Table 9: the number of task repetitions (NTR) within the given time, the task completion time (TCT), and the precision of the assembly process, as indicated by the standard deviation SD of the Euclidean distances between the assembled pieces. Participants subjectively rated their performance significantly higher in the visual-based instruction session. Participants seem to prioritize the possibility of repeating the task beyond its lower limit, disregarding the precision of their work. This higher repetition number gave them a sense of achieving their task with high performance in the visual-based session compared to the code-based session. The objective performance metrics aligned the subjective rate with respect to the NTR and TCT, as shown in Table 9 and Fig. 6a and b. In most cases, participants repeated the task significantly more during the visual-based session without exceeding the allocated time.

However, while the industry aims to increase production batches with short production times, it does not overlook the importance of product quality. In this study, the SD of the assembly process represented this metric. Due to their increased focus on the NTR, participants did not pay as much attention to their assembly precision. This resulted in a higher SD for the visual-based session, indicating lower precision compared to the code-based instruction session, where participants thoughtfully assembled each assembly piece without rushing through the process (See Fig. 6c). This suggests that while visual instructions may enhance speed and reduce perceived effort, they may inadvertently encourage less attention to detail. This objective metric is primarily not aligned with the subjective performance in the NASA_TLX. This contrast highlights the strengths and limitations of each measurement approach: subjective tools, such as NASA_TLX, can capture perceived workload or satisfaction, but they might miss more complex aspects of actual task performance. Objective measures like assembly precision provide quantifiable outcomes but do not fully account for internal states such as confidence or perceived effort.

The mismatch between objective metric and the subjective metric of performance might be understood within the framework of the Dynamic Model of Sustained Attention and Stress^65^. According to this model, individuals adjust their attention and effort allocation dynamically based on perceived task demands, available cognitive resources, and stress level. Thus, when task demands decrease, attention can become less focused, leading to a drop in task performance. Therefor, while the visual-based instruction optimize speed and effort, it may not sufficiently maintaine the level of attention needed for precision. These findings highlight the impratnce of achieving the optimal cognitive load during tasks. Although visual instruction can reduce cognitive load and increase assembly speed, it may not result in optimal cognitive performance in terms of precision. This indicates that while visual instruction may lower cognitive load and enhance efficiency, it might compomise attention and precision. Thus, in high-stakes or precision-demanding tasks, a certain level of cognitive load might be necessary to ensure attention and accurate performance. Therefore, instruction design should consider not just reducing cognitive load but also achieve optimal cognitive load that supports both efficiency and precision, optimizing overall task performance.

Future research can explore hybrid or modified methods to mitigate this trade-off. For instance, adaptive or context-sensitive instructions could primarily use visual aids for most assembly steps, yet incorporate code-based details during critical high-precision tasks. Alternatively, layered instructions-where a simple visual overview is supplemented by optional, more detailed code-based guidance-could preserve the clarity of visual methods while ensuring precision where it is needed. Such approaches might achieve a more optimal balance between efficiency and precision without overstressing the operator’s cognitive resources.

## Limitations and future research

This study faced several limitations that suggest directions for future research. One key limitation of this study is that the experiment was conducted in a controlled laboratory environment, which does not fully mirror the complexity of real-world industrial settings. In actual production lines, factors like noise, teamwork, multitasking, and real-time pressures can substantially influence the operators’ cognitive load and performance. Consequently, the results presented here should be interpreted as foundational insights rather than direct predictions of on-site outcomes. Nonetheless, our findings highlight the importance of minimizing extraneous cognitive load in designing effective work instructions. Future research may further validate these insights by integrating realistic workplace parameters-such as time constraints, loud machinery, and group-based tasks-into experimental protocols.

Another limitation of this study is the sample representativeness, where the participant pool was restricted to university students and researchers. While this homogeneous sample allowed for consistent baseline characteristics, it may not adequately represent the demographic and experiential diversity of industrial workers. Therefore, caution is warranted in generalizing our findings to actual industrial environments. In future work, we plan to broaden our sample to include operators from various industrial settings. This expanded approach will help validate our current results and further refine guidelines for optimal instructional design.

Additionally, physiological sensor placement on the non-dominant hand limited task execution to one-handed, which was another limiting factor in this study. This constraint potentially affected both the pace and strategies used, reducing the ecological validity of our findings. In future studies, we plan to adopt less intrusive sensor placements (e.g., wearable wristbands, arm and chest straps, or forehead sensors) to enable two-handed operation and better replicate industrial conditions.

Moreover, the study utilized a limited set of physiological signals (GSR and PPG). Incorporating a broader array of biosignals, such as eye tracking, body motion or posture tracking, electromyography (EMG), electroencephalography (EEG), and electrooculography (EOG), can provide deeper insights into the cognitive and physical states of workers, offer more robust support for the study’s hypotheses, or even provide a different point of view. Furthermore, while the sample size of 30 participants was substantial, future studies can expand it to enhance the statistical power and generalizability of the findings.

Finally, the five-minute time limit for the instruction sessions, which could only be extended if the specific pattern was not repeated three times, restricted most participants to completing the code-based session only three times. This prevented us from examining the full learning curve. It is possible that with more practice, participants could become more efficient with code-based instructions, potentially improving task performance over time.

## Conclusion

In this study, we found that visual-based instructions significantly reduce cognitive load and improve some operational aspects, such as shorter TCT and higher NTR compared to code-based instructions. However, our findings show a clear divergence between participants’ subjective ratings of performance through the NASA_TLX and the objective performance metric, assembly precision. While subjective measures are valuable for gauging perceived workload and emotional states, they can be influenced by factors like self-efficacy and momentary satisfaction. Conversely, the objective precision metric provides a direct measure of actual task outcomes but may overlook internal experiences of strain. As a result, high subjective performance scores did not always correspond to high objective precision.

Our study suggests that simple and direct instructions (visually based in this study) can enhance some of the operational aspects and reduce cognitive load, demonstrating that these kinds of instructional strategies are particularly beneficial in environments where quick task execution is critical. On the other hand, for tasks that require high precision and meticulous attention to detail, instructions that require deep thinking (code-based in this study) may be more appropriate. This discrepancy underscores the importance of a multi-method approach. Future research should explore more granular correlations between subjective and objective measures-perhaps by collecting in-task self-reports or by utilizing continuous physiological monitoring that can be compared against real-time performance logs. These insights can aid in developing customized training and operational protocols that improve productivity and enhance worker satisfaction.

## Data Availability

Data available upon request to Tamás Ruppert.
